# Effects of Icariin on Atherosclerosis and Predicted Function Regulatory Network in ApoE Deficient Mice

**DOI:** 10.1155/2018/9424186

**Published:** 2018-11-06

**Authors:** Yibing Zhang, Xiaoyan Ma, Xiangjun Li, Tong Zhang, Meng Qin, Liqun Ren

**Affiliations:** ^1^Department of Pharmacology and Toxicology, Jilin University School of Pharmaceutical Sciences, 1163 Xinmin Ave, Changchun, 130021, China; ^2^Department of Ophthalmology, First Hospital of Jilin University, Changchun, 130021, China; ^3^Department of Cardiology, The Affiliated Hospital of Changchun University of Chinese Medicine, 1478 Gongnong Ave, Changchun, 130021, China

## Abstract

*Objective. *Icariin plays a pivotal role in ameliorating atherosclerosis for animal models although its comprehensive biological role remains largely unexplored. This study aimed to fully understand the effects of icariin on atherosclerosis in high-fat diet-induced ApoE^−/−^ mice and investigate mRNA-miRNA regulation based on microarray and bioinformatics analysis.* Methods.* The areas of atherosclerotic lesions in en face aorta were evaluated. Microarray analysis was performed on atherosclerotic aortic tissues. The integrative analysis of mRNA and miRNA profiling was utilized to suggest specific functions of gene and supply an integrated and corresponding method to study the protective effect of icariin on atherosclerosis.* Results. *Icariin attenuated the development of atherosclerosis that the mean atherosclerotic lesion area was reduced by 5.8% (P < 0.05). Significant changes were observed in mRNA and miRNA expression patterns. Several miRNAs obtained from the miRNA-Gene-Network might play paramount part in antiatherosclerotic effect of icariin, such as mmu-miR-6931-5p, mmu-miR-3547-5p, mmu-miR-5107-5p, mmu-miR-6368, and mmu-miR-7118-5p. Specific miRNAs and GO terms associated with icariin in the pathogenesis of atherosclerosis were validated using GO analysis and miRNA-GO-Network. MiRNA-Pathway-Network indicated that icariin induced miRNAs mainly regulate the signaling pathways of PI3K/Akt signaling pathway, Ras signaling pathway, ErbB signaling pathway, and VEGF signaling pathway in aorta atherosclerotic lesion.* Conclusions. *Our data provides evidence that icariin is able to exhibit one antiatherosclerotic action by mediating multiple biological processes or cascades, suggesting the pleiotropic effects of icariin in atherosclerosis alleviation. The identified gene functional categories and pathways are potentially valuable targets for future development of RNA-guided gene regulation to fight disease.

## 1. Introduction

Atherosclerosis is characterized with marked structural alterations in the arterial wall induced by the sub-endothelial accumulation of modified lipoproteins followed by the chronic inflammatory response [1, 2]. It underpins many cardiovascular diseases with high mortality rates globally, such as stroke and coronary heart disease [3]. Atherosclerotic vascular remodeling is an adaptive response of the circulatory system to the growth of atherosclerotic plaques. The biomechanical and hemodynamic characteristics induce signaling cascades that contribute to angiogenesis [4, 5]. Indeed, atherosclerosis is a complex physiological process that results from the interactions of a range of factors. It is accompanied with upregulation or downregulation of various molecular subsets including microRNAs (miRNAs).

MiRNAs are short noncoding RNAs that regulate gene expression by mRNA degradation or translational repression [6]. Deregulated miRNAs have been associated with all kinds of human diseases; accumulating experimental evidence has revealed a key role for miRNAs in regulating cellular processes related to cardiovascular disorders. MiRNAs also play a prominent part in the pathogenesis of atherosclerosis, ranging from risk factors to plaque initiation and progression, up to atherosclerotic neovascularization [7–11]. Insights into miRNA-mRNA interaction are essential to understand the modulation of gene expression at the post-transcriptional level. High-throughput gene chip and bioinformatics technology offer unparalleled opportunities to address such issues and contribute to the current understanding of the complicated molecular mechanisms.

Although the application of statins was carried out over the past few decades [12], the high incidence of atherosclerosis-related cardiovascular disease still imposes an enormous burden on healthcare systems. Hence, novel concepts have been established in the treatment of atherosclerosis [13, 14]. Medicine herbs emerge as important sources of cardiovascular protective drugs that target multiple cellular processes of atherosclerosis [15]. Epimedium, a traditional oriental herbal medicine, is widely used to treat various diseases such as coronary heart disease, osteoporosis, and impotence [16, 17]. Icariin (ICA) (C_33_H_40_O_15_; molecular weight: 676.662 g/mol) is the main bioactive pharmaceutical constituent in Herba Epimedii. Evidence indicates that ICA possesses beneficial actions for protecting cardiovascular system, modulating lipid effects, stimulating osteoblast proliferation, and enhancing immune function [18–21]. Moreover, ICA has demonstrated eminent protects against atherosclerosis both in vivo and vitro models, which suggests its therapeutic potential in preventing or treating cardiovascular diseases [22–27]. However, few studies focused on the underlying RNA-mediated gene regulation of ICA. Especially, the modulations of ICA that occur in atherosclerotic animal model at the gene expression level and the interactions between miRNAs and mRNAs for the regulation of gene expression remain unknown.

In the context, this study was designed to explore the gene expression profiles as well as gene regulatory mechanisms of ICA on atherosclerosis. ApoE^−/−^ mouse has been an ideal model in amount of genome-wide transcriptional analysis. Thus, simultaneous miRNA and gene microarray profiling of the aorta of ICA-treated ApoE^−/−^ mice and model samples was performed to analyze the attendant transcriptome. By integrated analysis of the microarray results, our data provide more clues for the various biological role of the action of ICA against atherosclerosis and further indepth verifications are needed.

## 2. Materials and Methods

### 2.1. Animal Treatment

All animal experiments were carried out in accordance with the National Research Council's guidelines. This study was conducted in conformity with the policies and procedures of the National Institutes of Health guide for the care and use of Laboratory animals (NIH Publications No. 8023, revised 1978). Male ApoE^−/−^ mice of eight weeks old on C57BL/6J background were purchased from Beijing Huafukang bioscience Co., Inc., Institute of Laboratory Animal Science, Chinese Academy of Medical Sciences (Beijing, China, certified No. SCXK (Jing) 2014-0004). Each mouse was kept in the following conditions: 22 ± 2°C room temperature, 50 ± 10% humidity, 12-h light period, and 12-h dark period (8:00 am lighting, 8:00 pm off). All mice were fed a normal-chow diet during their one-week acclimatization period. Then twelve ApoE^−/−^ mice were randomly divided into two groups (six for administration of ICA and six for model). Accelerated atherosclerosis was induced by feeding the mice a high-fat diet (HFD) containing 1.25% cholesterol and 20% fat (D12108C, Research Diets Inc). Every morning at 9:00, each group was administered 0.2 ml ICA or carboxymethylcellulose sodium (CMC-Na) by gavage for 12 weeks. The 40 mg/kg/day of ICA was chosen as the dosage as described in our previous study [23].

### 2.2. Analysis of Atherosclerotic Lesions

After 12 weeks of HFD feeding, mice were euthanized and perfused with 20 ml phosphate-buffered saline (PBS) through the left ventricle, and then fixed with PBS containing 4% paraformaldehyde. The heart and aortic tissues were then removed from the aortic root to the iliac bifurcation and placed in 4% paraformaldehyde for 24 h. Oil red O staining was performed to evaluate lipid and plaque accumulation in the aorta following the protocol published before [28]. To evaluate atherosclerotic lesion areas, the aorta was cut longitudinally to expose the intimal surface. Lesions in the aortic area were quantified from the aortic arch to the iliac bifurcation. Images were captured by a Canon EOS760 IS digital camera (Tokyo, Japan). Quantitative analysis of positive staining areas was done using Image-Pro Plus 6.0 software (NIH Image, USA). The lesion area of aortic atherosclerosis was expressed as the percentage of Oil Red O-stained area relative to the surface area of the entire aorta. All researchers conducting the data capture and analysis were blinded to group.

### 2.3. Microarray Analysis

For Affymetrix® microarray profiling, the total RNA was isolated from three replicate samples of icariin (40 mg/kg) and model groups using TRIzol reagent (Invitrogen, Carlsbad, Canada) and purified with an RNeasy Mini Kit (Qiagen, Hilden, Germany) according to the manufacturer's protocol. The quality and amount of RNA were measured using a UV-Vis Spectrophotometer (Thermo, NanoDrop 5000, USA) at an absorbance of 260 nm and RNA integrity was determined by gel electrophoresis.

Expression profiling of mRNA was determined by Clariom™ D solutions for mouse (Affymetrix GeneChip, Santa Clara, CA) and the miRNA expression profiling was measured by GeneChip® miRNA 4.0 Array (Affymetrix GeneChip, Santa Clara, CA). The microarrays were performed by Cnkingbio, which is accredited by Affymetrix®. The microarray analysis was manifested using Affymetrix® Expression Console Software (version 1.2.1). Raw data (CEL files) were normalized at the transcript level using the robust multi-array average method. The median summarization of transcript expressions was calculated. The gene-level data were then filtered with the probe sets in the Bcore∧ meta-probe list which represents RefSeq genes.

### 2.4. Bioinformatics Analysis

#### 2.4.1. Differential Expression of mRNAs and miRNAs

The random variance model (RVM) t-test was used to filter differentially expressed mRNAs and miRNAs (DE mRNAs, DE miRNAs) for the treatment and model groups. To discern the genes and miRNA that are differentially expressed, we chose a p value < 0.05 by ANOVA as the threshold screening between the treatment and the model groups. Hierarchical clustering of mRNAs and miRNAs with significantly different expression was processed by the Cluster 3.0 software and visualized with Treeviewv1.60 (Cnkingbio, Beijing, China). The expressed data was normalized by using the median summarization method.

#### 2.4.2. Prediction of Targeted Gene and Construction of MicroRNA-Gene-Network

The miRNA target prediction tools, TargetScan and miRnada, were utilized to further explore the targeted genes, which were regulated by differentially expressed miRNAs. Prior to the prediction of the mRNA targets of these miRNAs based on the TargetScan and miRanda, consistently upregulated or downregulated miRNAs were defined. Subsequently, an intersection between the targets of miRNAs and differentially expressed genes (DE genes) was acquired. The regulatory relationships between miRNAs and genes were obtained related to their expression values and the microRNA-gene-network was built based on their interactions [29].

#### 2.4.3. Go Analysis and MicroRNA-GO-Network

Gene Ontology (GO) analysis was applied to analyze the major function of the specific genes with significant differences in the representative profiles of DE genes. GO analysis can uncover the gene regulatory network on the basis of biological processes and molecular function and organize genes into hierarchical categories [30]. Specifically, the chi-square test and two-side Fisher's exact test could classify the GO category. Enrichment provides a measure of the significance of the function: as the enrichment increases, the corresponding function is more specific, which helps us to find those GOs with more concrete function description in the experiment. The microRNA-GO-network was established based on the relationships between significant GO categories and genes and the relationship between miRNA expressions and gene expression [31–33].

#### 2.4.4. Pathway Enrichment Analysis and MicroRNA-Pathway-Network

Pathway analysis could locate the significant pathways of the DE genes according to KEGG (Kyoto Encyclopedia of Genes and Genomes). Significant pathways were selected by Fisher's exact test and chi-square test [34] and the standard of difference was p < 0.05. The microRNA-pathway-network was built according to the relationships among significant pathways and genes/miRNAs.

### 2.5. Statistical Analysis

Statistics in microarray and bioinformatic analyses were performed by one-way analysis of variance (ANOVA) using Affymetrix® Expression Console™ TAC (Affymetrix® Expression Console™), followed by the least significant difference (LSD) test; statistics of atherosclerotic lesion area were performed by one-way ANOVA followed by Student's t-test, using SPSS 16.0 software (SPSS, Chicago, IL, USA). Significant differences were considered at p < 0.05.

## 3. Results

### 3.1. Icariin Reduces Atherosclerotic Lesion Area in ApoE^−/−^ Mice

**Figure 1:**
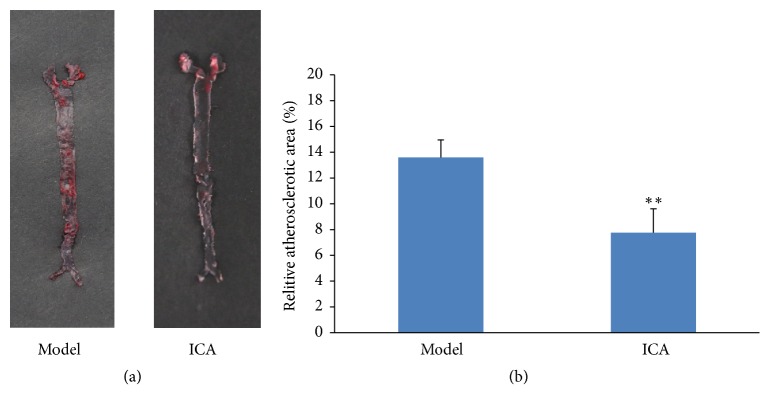
Effect of icariin on atherosclerotic plaque area in HFD-fed ApoE^−/−^ mice. (a) The atherosclerotic lesions of each group of mice were stained by Oil Red O. ApoE-/- mice were treated with 40mg/kg/day icariin (ICA) or carboxymethylcellulose sodium (Model) for 12 weeks. (b) Plaque area was quantified as the percentage of Oil Red O-stained area to total aortic surface area. Values are expressed as means ± SEM. ^*∗∗*^P<0.05 vs. Model.

The aortas from high-fat diet treated ApoE^−/−^ mice exhibited atherosclerotic plaques that were readily visible in the model group. Atherosclerotic plaques developed in the aortic root and were sporadically present throughout the aorta, and extensive and severe lesions were identified in the aortic roots especially. En face analysis revealed that ICA treatment with 40 mg/kg per day significantly reduced the size of Oil Red O-stained atherosclerotic plaques in the aortas by 5.8% (Figures 1(a) and 1(b)).

### 3.2. DE Genes and DE miRNAs in the Effect of Icariin

**Figure 2:**
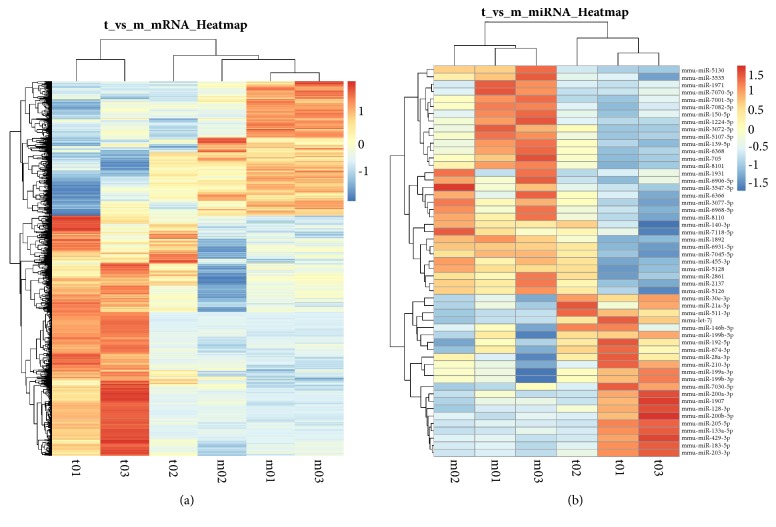
Hierarchical cluster analysis in apoE deficient mice administrated with icariin. (a) Differential expression of mRNA. (b) Differential expression of microRNA. p-values < 0.05. The relative gene log⁡2 expression changes are expressed by a color gradient intensity scale. Blue color indicates downregulation, and red color indicates upregulation of mRNAs or miRNAs expression. Each row represents a single mRNA or miRNA and each column represents a separate sample.

From the microarray analysis, mRNA and miRNA expression profiles of ApoE^−/−^ mice administrated to ICA were obtained. Compared with the model groups, there were a total of 2059 DE genes and 53 DE miRNAs based on nominal p-values<0.05 triggered by ICA treatments (Figures 2(a) and 2(b)).

### 3.3. Potential Targets Genes of DE miRNAs and MicroRNA-Gene-Network

An intersection between the targeted genes of miRNAs based on the TargetScan and miRnada was obtained. Thus, 51 miRNAs (the other miR-1892 and miR-1907 were not available in the Targetscan database) and 3932 target genes were acquired in total. Furthermore, four hundred and eighteen genes were obtained with the intersection of 3932 target genes and 2059 differentially expressed genes. MiRNAs regulate gene expression via degradation or translational inhibition of their target mRNAs [35]. Consequently, the mRNAs which expressions were negatively correlated with miRNAs expression were acquired finally.

**Figure 3:**
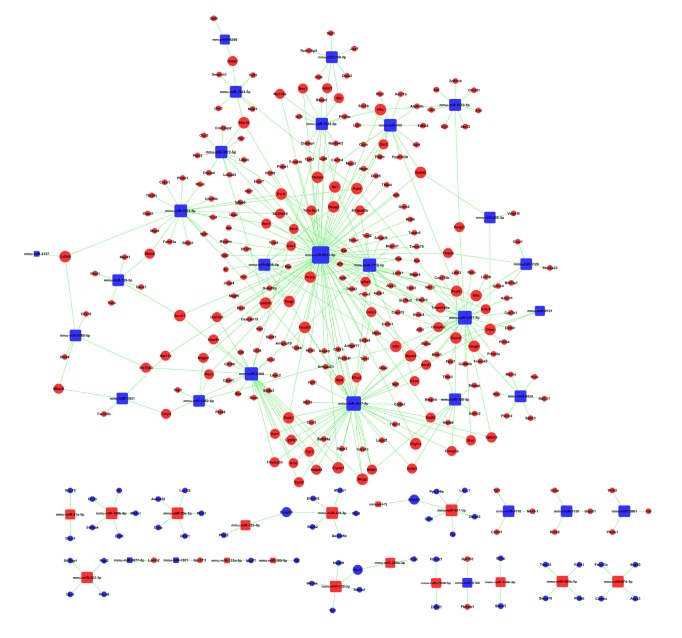
miRNA-gene-network. Box nodes represent miRNAs, and cycle nodes represent the predicted target genes. Edges show the inhibitory effect of miRNA to its predicted targets. Degree means the contribution one miRNA to the genes around or the contribution one gene to the miRNAs around. The key miRNAs and genes in the network always have the highest degrees.

Since the negatively regulatory relationships between miRNAs and genes were gained, the microRNA-gene-network was constructed based on reverse regulated miRNA-mRNAs, which included 46 miRNAs and 309 mRNAs. The top 10 miRNAs ranked by degree over 10 were mmu-miR-6931-5p, mmu-miR-3547-5p, mmu-miR-5107-5p, mmu-miR-6368, mmu-miR-7118-5p, mmu-miR-7001-5p, mmu-miR-705, mmu-miR-7045-5p, mmu-miR-150-5p, and mmu-miR-3072-5p, which were found to have more remarkable effects and may have vital regulatory effects in the network (Figure 3).

### 3.4. GO Analysis and MicroRNA-GO-Network

**Figure 4:**
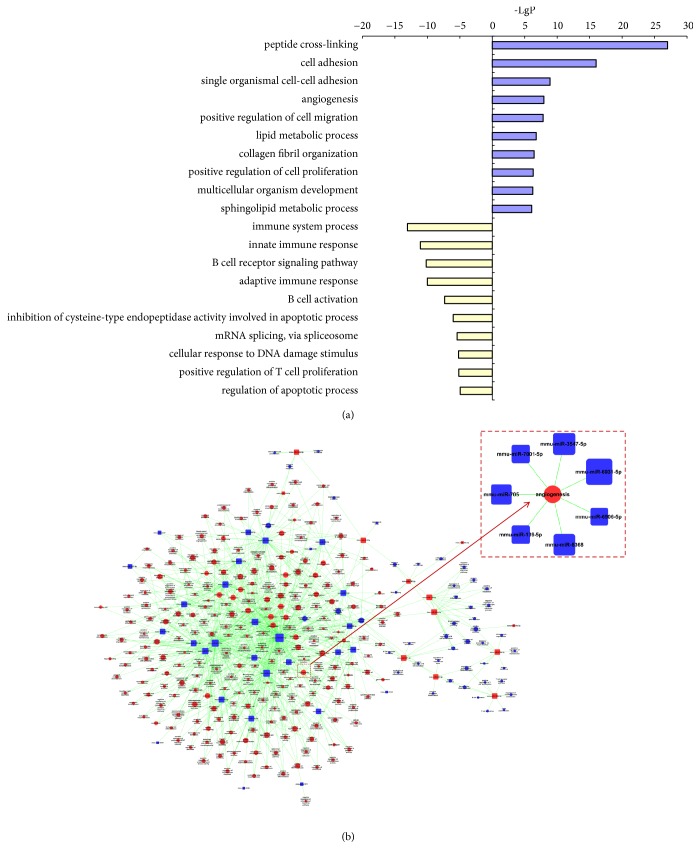
GO analysis and MicroRNA-GO-network. (a) GO analysis based on DE genes. The vertical axis is the GO category, and the horizontal axis is the enrichment of GO. It shows significant changes in gene function, upregulation GO (-lg P) and downregulation GO (lg P), respectively. (b) MicroRNA-GO-network. Red box nodes represent upregulated miRNA, blue box nodes represent downregulated miRNA, red cycle nodes represent upregulated GO, and blue cycle nodes represent downregulated GO. Edges show the effect of microRNA on GO. The sub-network is extracted from the whole microRNA-GO-network.

GO analysis was carried out to analyze the significant gene function. The representative profiles of DE genes were shown in Figure 4(a). On the biological process level, the upregulated DE genes were enriched in GO terms such as peptide cross-linking, cell adhesion, single organismal cell-cell adhesion, angiogenesis, positive regulation of cell migration, and lipid metabolic process. The downregulated DE genes were enriched in GO terms such as immune system process, innate and adaptive immune response, and apoptosis process level in response to ICA administration.

To reveal miRNA regulation of annotating gene function, miRNA-GO network was built. 286 significant GO terms and 41 miRNAs were identified; the related target genes were significantly enriched in the GO annotations ‘multicellular organism development, protein phosphorylation, apoptotic process, transport, cell differentiation, metabolic process, cell adhesion, cell proliferation, cell migration, cell adhesion, and angiogenesis.' The top five miRNAs were mmu-miR-6931-5p, mmu-miR-3547-5p, mmu-miR-6368, mmu-miR-705, and mmu-miR-7118-5p, which were involved with a large number of GO annotations (degree > 50). The network concisely showed the relationship between the miRNAs and their related target gene function (Figure 4(b)). Moreover, the subnetworks suggested that sets of genes worked together to accomplish the multistep processes, such as angiogenesis.

### 3.5. MicroRNA-Pathway-Network

**Figure 5:**
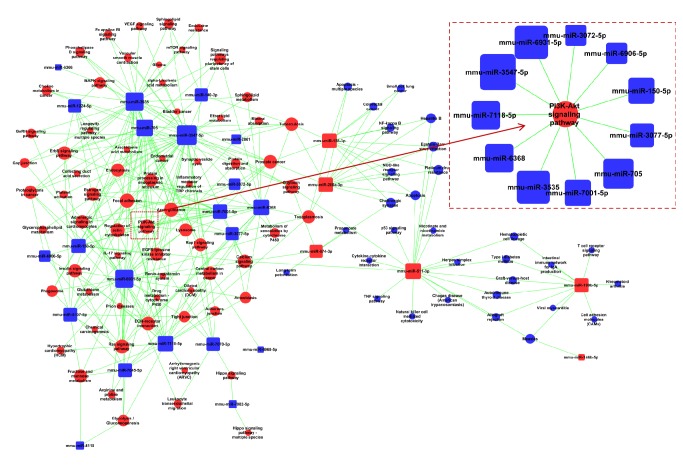
MicroRNA-pathway-network. Red box nodes and blue box nodes represent upregulated miRNA and downregulated miRNA, respectively. Blue cycle nodes represent Pathway. Edges show the inhibitory effect of miRNA on Pathway. When the area of box or circle is larger, the degree of the miRNA or pathway is bigger. The sub-network extracted from the whole network includes the key miRNAs and pathways.

The significant pathways were discerned in accordance with the functions and interactions of DE genes derived from KEGG database. Based on the significantly regulated pathways, we further established miRNA-pathway networks to screen the key regulatory functions and the key DE miRNAs (Figure 5). There were 104 significant differentially expressed pathways and 27 miRNAs included. The DE mRNAs mainly play essential roles in various biological processes; the top 10 pathways were involved in such as signal transduction (PI3K/Akt signaling pathway, Ras signaling pathway, ErbB signaling pathway, and VEGF signaling pathway), signaling molecules and interaction (ECM-receptor interaction), cellular process (Focal adhesion, regulation of actin cytoskeleton, and endocytosis), and organismal systems (Axon guidance and insulin signaling pathway). The top rated seven miRNAs included mmu-miR-3547-5p, mmu-miR-6931-5p, mmu-miR-3535, mmu-miR-705, mmu-miR-511-3p, mmu-miR-6368, and mmu-miR-7118-5p (degree > 15). Taken together, ICA exerted to target specific signaling cascades in ApoE^−/−^ mice to generate antiatherogenic properties. These networks provided a large amount of information and systematically analyzed the signaling cascades mediating protective effects of ICA.

## 4. Discussion

Previously, we and others have demonstrated that ICA attenuates atherosclerosis potently [22–27]; nevertheless, the concrete regulatory mechanism remains to be understood. Due to the biological complexity of atherosclerosis, comprehensive understanding of the specific role of ICA in cardiovascular disease is meaning for treat strategy. In this study, we extracted useful data and translate them into mechanistic understandings by different bioinformatic approaches. The integrated analysis of miRNA-mRNA interaction was utilized to help us improve the cognition of gene regulations on antiatherosclerotic effect of ICA.

The hallmark feature of atherosclerosis is the formation of plaque, which is the build-up of fatty deposits, debris, and cellular components underneath the inner wall of the blood vessel. In this work, we observed that 12-week treatment with the ICA remarkably reduced the lesion area by 5.8% in the ApoE^−/−^ mice fed with HFD. This reduction of plaque formation in ApoE^−/−^ mouse model was consistent with a previous study [36]. ApoE^−/−^ mouse is a well-established animal model for studying atherosclerosis. ApoE^−/−^ mice feeding normal chow could spontaneously develop atherosclerotic lesions, which are provided with the HFD developed more advanced lesions. Fatty streaks in the proximal aorta are found at 3 months of age [37]. Moreover, we used ApoE^−/−^ mouse model in the present study since its pathogenesis of atherosclerosis resembles that observed in humans and it has been widely applied in cardiovascular research.

Atherosclerosis-associated obstruction of large- or medium-sized arteries in the myocardium and brain is the most common cause of lethal human diseases, for which minimal therapeutic strategies exist. Thus, prevention and treatment of ischemia diseases has become a significant goal and great efforts have been undertaken to implement tissue regeneration strategies using proangiogenic agents, such as vascular endothelial growth factor (VEGF). Angiogenesis is a fundamental step in a variety of physiological and pathological conditions. Broad and complex biological process actions are implicated in angiogenesis, such as endothelial cell proliferation, migration, and differentiation. Of all the differentially expressed genes, a substantial proportion enriched in the GO annotations were related with neovascularization, including angiogenesis, cell migration and cell proliferation on the biological process level. This is consistent with the reported proangiogenic properties of ICA. Tang et al. found that ICA obviously stimulated cell migration and induced angiogenic differentiation in endothelial progenitor cells (EPCs). Furthermore, they herein found that ICA modulated angiogenesis by enhancing vasculogenesis and protecting against endothelial dysfunction both [38]. Moreover, Jing et al. demonstrated that VEGF protein expression was significantly increased after ICA administration, ICA definitely promoted the angiogenesis of rat EPCs and this capacity was inhibited by a VEGF/VEGF receptor-specific binding inhibitor bevacizumab [39]. Paradoxically, in vitro evidence also indicated that ICA may directly stimulate angiogenesis, without affecting VEGF expression in human umbilical vein endothelial cells (HUVECs) [40]. Besides, Chung et al. also showed that ICA possessed potent angiogenic activity through the activation of the MEK/ERK and PI3K/Akt/NO-dependent signal pathways, which was in line with the results of Koizumi et al [41]. In addition, further studies were performed to clarify the above involving signal pathway. Interestingly, Duan et al. suggested that PI3K/Akt/NO-dependent signaling pathway might be responsible for this efficacy of ICA, but MEK/ERK did not show this ability [42]. Therefore, the mechanism of ICA related to angiogenesis needs more experimental explorations.

Pathway-based analysis is a useful tool to detect changes at a higher biological level than individual genes, complementing single gene level approaches with biological interactions and thus contributing to a better understanding of the complexity of diseases [43]. Identification of dysregulated pathways is proposed as possible biomarkers for such multifactorial disorders. Our results confirmed that the core pathway involved in the positive response to ICA was PI3K/Akt signal pathway, derived from the miRNA-pathway-network (degree = 11) (Figure 5). Serine/threonine protein kinase AKT, also known as protein kinase B, is stimulated by a number of receptor tyrosine kinases and G protein-coupled receptors through phosphatidylinositol 3-kinase (PI3K). AKT protein kinase plays an important role in a number of biological processes including metabolic responses, proliferation, differentiation, and growth [44, 45]. Surprisingly, PI3K/Akt signal pathway was one of the most central nodes in our network and it was described as a potential pathway associated with ICA response in the literature. This showed that our network analysis was able to capture the prominent signal pathways in ICA treated ApoE^−/−^ mice. Further studies are required to investigate how ICA activates the phosphorylation of AKT, or which signal molecules lies in the upstream of PI3K/Akt signal pathway.

MiRNAs are estimated to regulate more than 60% of whole human protein-coding genes, act as safeguards for the cell, and exhibit essential regulatory functions related to cell growth, apoptosis, development, and differentiation. MiRNA and mRNA expression negative associations can be identified if the mRNA is degraded after being targeted [46, 47]. Importantly, each miRNA can modulate the expression of multiple mRNAs and each mRNA may be targeted by several different miRNAs, leading to complex regulatory networks which are poorly characterized. Likewise, there has been much effort to define patterns of miRNAs dysregulation that could aid in the diagnosis and prognostication of human diseases. Furthermore, the role of miRNA in therapy is also an emerging field. Cumulative studies have demonstrated that miRNA patterns could be altered in drug treatment. Recently, miRNAs have emerged as key regulators of atherosclerosis in ApoE^−/−^ mice [48, 49]. Our results suggest a possibility of miRNAs mediating the beneficial effects of ICA with core signaling pathways. We have set up miRNA-pathway-network to implicate several miRNAs as a regulatory ‘‘hub” involved in posttranscriptional silencing of target genes. There were 27 DE miRNAs in the miRNA-pathway-network which potentially regulate the expression of genes associated with the effects of ICA in ApoE^−/−^ mice, but few of them have been reported. Multiple miRNAs regulated several overlapping targets among DE genes. The significant DE genes of PI3K/Akt signal pathway related to ICA were targeted by 11 DE miRNAs: mmu-miR-3547-5p, mmu-miR-6931-5p, mmu-miR-3535, mmu-miR-705, mmu-miR-6368, mmu-miR-7118-5p, mmu-miR-7001, mmu-miR-150-5p, mmu-miR-3077, mmu-miR-3072-5p, and mmu-miR-6906 (Figure 5).

GO analysis, is the classical method to organize differentially expressed genes into hierarchical categories based on gene function [50]. Coupled with the parallel results in miRNA-GO-network, we found that 7 DE miRNAs were directly correlated to angiogenesis, including mmu-miR-6931-5p, mmu-miR-3547-5p, mmu-miR-6368, mmu-miR-705, mmu-miR-7001-5p, mmu-miR-6906, and mmu-miR-139-5p. Meanwhile, the top three dysregulated miRNAs (mmu-miR-6931-5p, mmu-miR-3547-5p, and mmu-miR-6368) in miRNA-GO-network contributed to cell differentiation and migration also, which linked with angiogenesis (Figure 4(b)).

Collectively, these data suggest that angiogenesis, including the affected processes and pathways involved in beneficial actions of ICA on atherosclerosis are governed by some key miRNAs. The above miRNAs may provide novel biomakers for atherosclerosis progression useful in development of future targeted therapies. ICA is able to exhibit one antiatherosclerotic action by mediating multiple miRNAs, suggesting the pleiotropic and multitargeted effects of ICA in atherosclerosis alleviation.

Apart from the significant pathways in miRNA-pathway-network described above, multiple well-known pathways were dysregulated in ICA treatment group, compared with model group. There are a number of pathophysiological conditions in which these involving pathways plays a major role. For example, Focal adhesion, regulation of actin cytoskeleton, and ECM-receptor interaction were significant differentially expressed, which were in agreement with several reported bioinformatics analyses of gene expression associated with osteopathia. As mentioned in the literature, Epimedium has been used to exert bone protective roles for thousands of years in China, Korea, and Japan. As the main active flavonoid glucoside ingredient isolated from Herba Epimedii, more recent studies showed that ICA enhances osteogenic differentiation and inhibits osteoclast formation [51, 52]. Interestingly, some evidences of functional and pathway enrichment analysis showed that the dysregulated ECM-receptor interaction may be responsible for fracture healing and bone remodeling [53, 54]. In addition, Li et al. identified differentially expressed proteins and found that ECM-receptor interaction, focal adhesion, and regulation of actin cytoskeleton signaling pathway were involved in chondrocyte differentiation [55]. These publications in line with our study suggested single pathways to be involved in therapeutic effect of ICA. Signaling network motifs are a group of interacting genes acting in the network together and are capable of signal processing. They bear specific regulatory properties and mechanisms as seen in biological network studies [56]. Therefore, the network of miRNA-pathway interaction could be of a great tool for researchers who are interested in the mechanisms behind beneficial effects of ICA, since genes enclosed in the pathways could be new targets to investigate mechanism as well as possible future treatments.

The major limitation of our study is that the present results consisted of a set of network communities related with antiatherosclerotic effect of ICA. However, only a few subnetworks were relatively rich in information. Comprehensive information for other network communities was still unavailable. In addition, based on bioinformatics analysis, we filtered several miRNAs which were in the center of the regulatory network and corresponding target genes, such as mmu-miR-6931-5p. The key miRNAs which regulate the expression of genes were associated with the protective effects of ICA in ApoE^−/−^ mice, but few of them have been reported. Taking all these into consideration, further verifications and reasonable analyses are worthwhile to vindicate the relations between these miRNAs and antiatherosclerotic effect of ICA. Another limitation is that this present study obtained the meaningful miRNAs by enrichment analysis of the predicted miRNA targets, which might lead to unsatisfactory quality of miRNA sets and biased analysis outcomes [57]. Despite most of the current tools for miRNA functional annotation being based on target genes mainly, it is increasingly necessary to integrate miRNAs into groups directly based on miRNA annotations. Some miRNA set enrichment analyses represent the important tools for this purpose, such as miSEA, miEAA, and TAM [58–60]. These updated servers may be alternative tools for processing outputs of high throughput miRNA studies. In this sense, it should be the future direction to develop more advanced miRNA enrichment tools for providing more miRNA resources.

Increasing studies have been executed to identify potential treatment strategies for complex diseases which are caused by a collective dysregulation of many genes. Of note, specific high-throughput analyses of gene regulation have been the focus of numerous research studies. Substantial efforts have been made in this direction recently and have generated interesting results. Despite their broad biological involvement and challenges to elucidate specific function and mechanisms, detailed network analyses have become a valuable tool for gene interactions and screening processes in certain conditions. For instance, hallmark networks based on gene expression have been used to cancer prediction and drug target testing [61, 62]. Furthermore, early studies exemplified how to combine high-throughput data and bioinformatics algorithms to identify miRNA mediated regulatory motifs or to establish high-confidence integral miRNA-regulated networks [63–65]. Some papers provided experimental support that the miRNA-mRNA target relationships appeared to coordinately modulate multiple members of critical pathways associated with human cell biology [66–68]. Some other publications concentrated on the miRNA-mediated mechanisms of human metabolism and not only defined the interactions between miRNA pairs but also jointed the functional patterns shared by them. In particular, the network models could simplify the complexity of miRNA interactions by connecting them through their common pathways [69]. Taking together with the high reliability of the microarray technology nowadays, function and pathways analysis based on the microarray data can be considered as remarkably robust studies. Thus, the integrative network analysis of the miRNA-mRNA interaction is a reliable strategy to enhance the understanding of gene regulation. Actually, the comprehensive analyses in our result could show a clear expression pattern for DE genes and DE miRNAs that contribute to the vascular protective activity of ICA in ApoE^−/−^ mice. The networks could be of a great tool for researchers who are interested in the biological functions behind antiatherosclerotic effect of ICA, since genes enclosed in the GO terms and pathways could be new targets to investigate mechanism as well as possible future treatments. Our study conferred a strong evidence for ameliorating effect of ICA in the pathogenesis of atherosclerosis, which could support drug design in a more effective manner facilitating a more targeted medicine in patients with atherosclerosis.

## 5. Conclusion

In summary, our study shows that ICA could potently attenuate atherosclerosis and cause marked changes in gene and miRNA expression patterns in ApoE^−/−^ mice. Our data provide a comprehensive analysis of the genome-wide transcriptional changes that are induced by ICA. The most impact gene functional categories caused by ICA were angiogenesis, cell differentiation, and migration. PI3K/Akt signaling pathway was a critical pathway by treatment with ICA in ApoE^−/−^ mice. It supplies compelling clues for core function which was meaningful for reveal the regulation from miRNAs to mRNAs.

## Data Availability

The data used to support the findings of this study are available from the corresponding author upon request.

## Abbreviations

ICA: Icariin
HFD: High-fat diet
miRNA: MicroRNA
DE mRNAs: Differentially expressed mRNAs
DE miRNAs: Differentially expressed miRNAs
DE genes: Differentially expressed genes.
